# Supplementary material for: Deep Learning for Fluorescence Lifetime Predictions Enables High-Throughput In Vivo Imaging
Source: J Am Chem Soc. 2025 Jun 14;147(26):22609–21. doi: 10.1021/jacs.5c03749 (PMC12232305; doi:10.1021/jacs.5c03749)
Supplement: Supplementary file 1 [file ja5c03749_si_001.pdf]

Deep Learning for Fluorescence Lifetime Predictions Enables High-Throughput In Vivo Imaging

Sofia Kapsiani[1], Nino F. Läubli[1], Edward N. Ward[1], Ana Fernandez-Villegas[1], Bismoy Mazumder[1], Clemens F. Kaminski[1], Gabriele S. Kaminski Schierle[1,*]

[1] Department of Chemical Engineering and Biotechnology, University of Cambridge, Cambridge, CB3 0AS, UK

* Corresponding author: gsk20@cam.ac.uk

Contents

Supporting Figure 1. FLIMngo outperforms decay curve fitting on simulated data.......................... 2

Supporting Figure 2. FLIMngo can accurately analyse samples exhibiting multiple fluorescent components.......................... 3

Supporting Figure 3. FLIMngo successfully detects autofluorescence in *C. elegans*.......................... 4

Supporting Figure 4. FLIMngo reliably predicts simple exponential decays acquired with detectors having even very wide IRFs.......................... 5

Supporting Figure 5. FLIMngo is capable to analyse time-gated FLIM data that exhibit an exponential-like fluorescence decay.......................... 6

Supporting Figure 6. Predicted FLIMngo fluorescence lifetimes for mScarlet expressing *C. elegans*.......................... 7

Supporting Figure 7. Predicted phasor fluorescence lifetimes for mScarlet expressing *C. elegans*.......................... 8

Supporting Figure 8. Methodology for simulating TCSPC-FLIM data using the HPA dataset.......... 9

Supporting Figure 9. Simulated IRFs and their comparison to in-house data.......................... 10

Supporting Figure 10. Complete set of IRFs used in the simulation of FLIM data.......................... 11

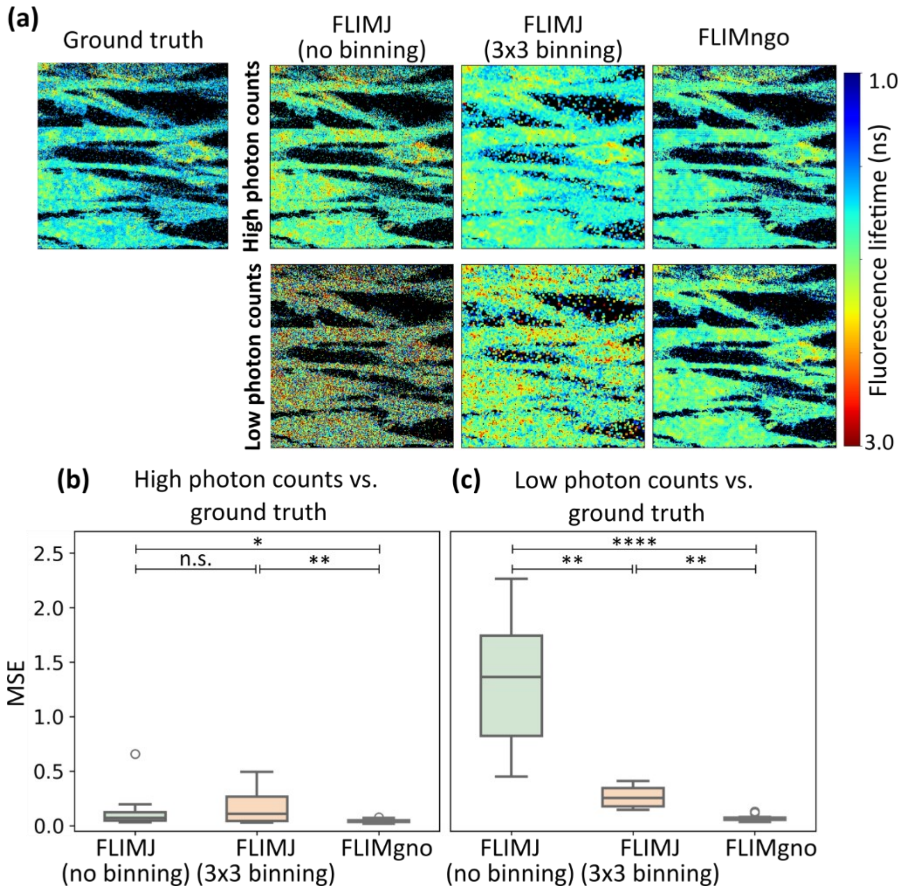

Supporting Figure 1. FLIMngo outperforms decay curve fitting on simulated data. (a) Fluorescence lifetime maps of the ground truth alongside the maps predicted by FLIMJ[1] (no pixel binning), FLIMJ (3x3 spatial pixel binning), and FLIMngo. The high and low photon count datasets have identical ground truth fluorescence lifetime maps. **(b)** Box-and-whisker plots of the MSE scores for predicted fluorescence lifetime maps from high photon counts compared to ground truth data. **(c)** Box-and-whisker plots of the MSE scores for predicted fluorescence lifetime maps from low photon counts compared to ground truth data. For the Box-and-whisker plots the line indicates the median, while the box represents the interquartile range; whiskers extend to the furthest data points within 1.5 times the interquartile range and the dots show outliers. The data consisted of 16 simulated images with high photon counts (100-2500 photons per pixel) and the same images with low photon counts (25-100 photons per pixel), respectively. Statistical significance was calculated using a Kruskal-Wallis test followed by Dunn's multiple comparisons, where * denotes $p < 0.05$, ** denotes $p < 0.01$, **** denotes $p < 0.0001$, and "n.s." denotes non-significant comparisons.

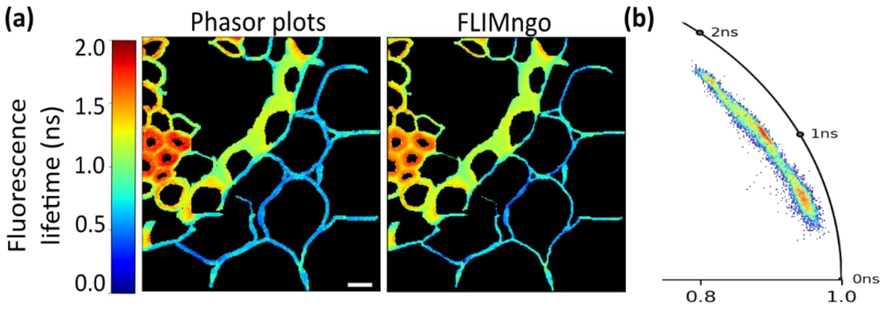

Supporting Figure 2. FLIMngo can accurately analyse samples exhibiting multiple fluorescent components. (a) Comparison between the phase lifetime map (left), obtained from phasor plot analysis, and the fluorescence lifetime map predicted by FLIMngo (right) for a *Convallaria rhizome* sample. The MSE score between the two lifetime maps is 0.007, indicating a high level of agreement. **(b)** Phasor plot of the *Convallaria rhizome*, highlighting the presence of complex fluorescence decay behaviour. The scale bar is 10 µm.

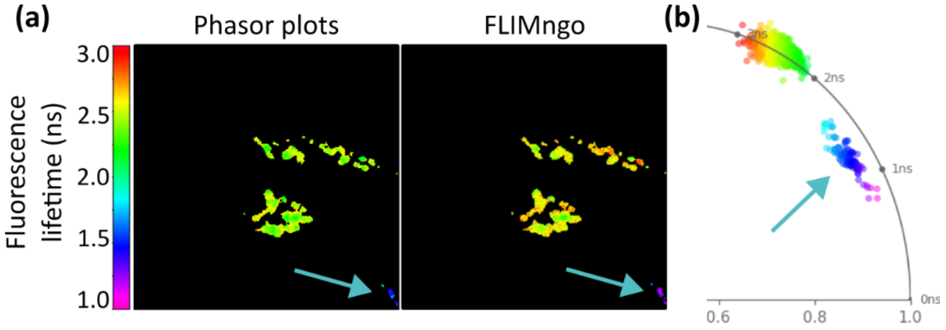

Supporting Figure 3. FLIMngo successfully detects autofluorescence in *C. elegans*. (a) Fluorescence lifetime maps showing *C. elegans* neurons expressing GFP-tagged FUS generated through phasor plot analysis (left) and FLIMngo (right). For the phasor plot data, the average of the phase and modulation lifetime is reported. **(b)** Corresponding phasor plot showing a separation between the GFP and autofluorescence cluster. Regions of autofluorescence are indicated by light blue arrows.

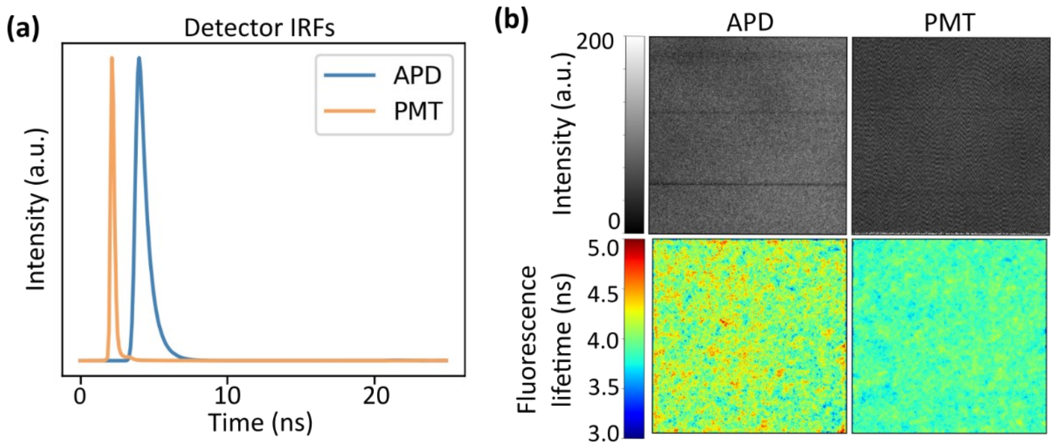

Supporting Figure 4. FLIMngo reliably predicts simple exponential decays acquired with detectors having even very wide IRFs. (a) IRFs obtained using the APD (blue) and PMT (orange) detectors. **(b)** Intensity images and predicted fluorescence lifetime maps for images captured with the APD (left) and PMT (right) detectors.

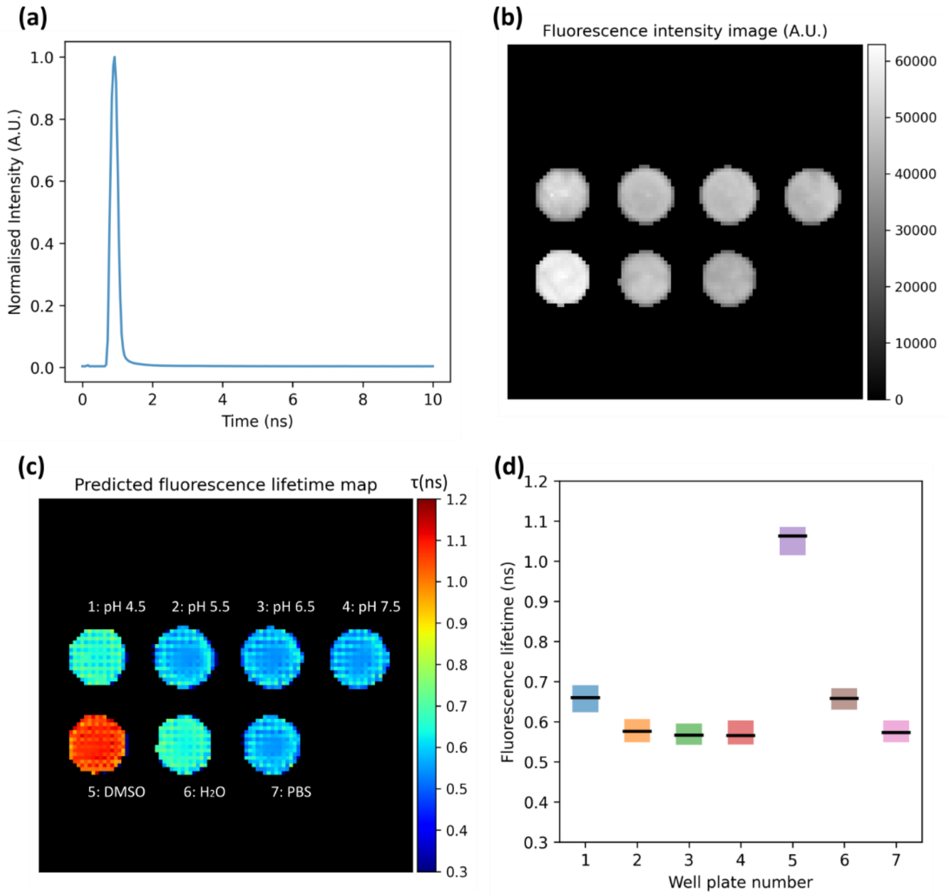

Supporting Figure 5. FLIMngo is capable to analyse time-gated FLIM data that exhibit an exponential-like fluorescence decay. FLIMngo was applied to analyse data collected with a gated intensified charge-coupled device (ICCD) camera, published by Smith et al. (2022)[2]. Specifically, dye solution of IRDye 800CW conjugated to 2-deoxyglucose were imaged either in buffers with different pH, DMSO, water or phosphate-buffered saline (PBS). **(a)** IRF from gated-ICCD system illustrating that the data exhibit an exponential-like fluorescence decay. **(b)** Fluorescence intensity map highlighting the high photon count content of the data, which is beyond the photon count range of the training data (10-2,500 photons per pixel). **(c)** FLIMngo-predicted fluorescence lifetime map of the IRDye 800CW data. The image has been edited in Inkscape to indicate the well-plate numbers and pH environments. **(d)** Box plots showing the predicted fluorescence lifetimes of each well. The fluorescence lifetimes predicted are in close agreement and follow the same trend with the nonlinear least squares fit (NLSF) analysis presented in the referenced study.

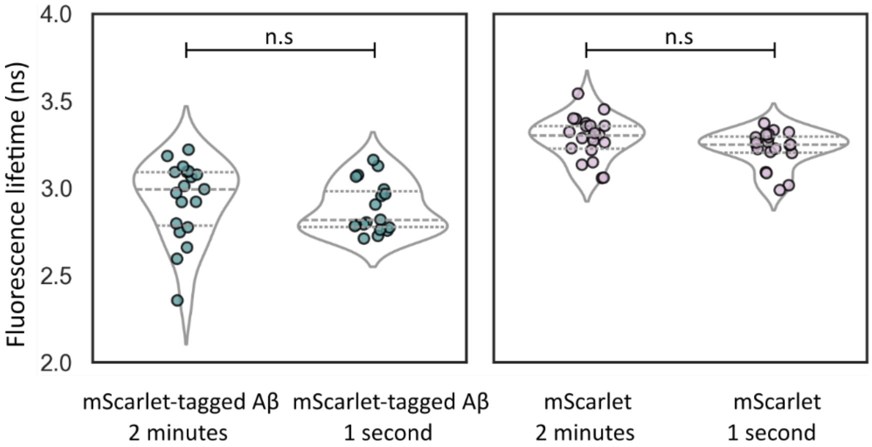

Supporting Figure 6. Predicted FLIMngo fluorescence lifetimes for mScarlet expressing *C. elegans*. Violin plots show FLIMngo predicted fluorescence lifetimes of neuronally expressed mScarlet tagged to $A\beta_{1-42}$ acquired in 1-second and 2-minute recordings as well as mScarlet without $A\beta_{1-42}$ imaged in 1-second and 2-minute recordings. The significance levels have been calculated using the Mann–Whitney U test, where "n.s" denotes not significant.

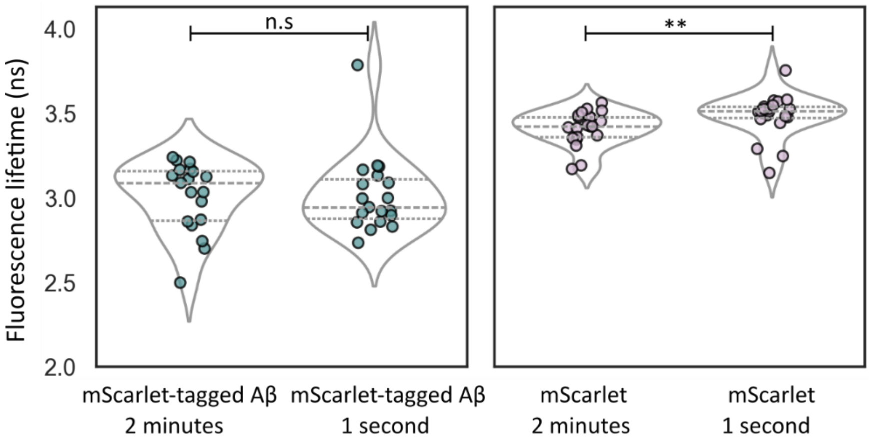

Supporting Figure 7. Predicted phasor fluorescence lifetimes for mScarlet expressing *C. elegans*. Violin plots showing phasor fluorescence lifetimes of neuronally expressed mScarlet tagged to A$\beta_{1\text{-}42}$ acquired in 1-second and 2-minute recordings as well as mScarlet without A$\beta_{1\text{-}42}$ also imaged in 1-second and 2-minute recordings. The significance levels have been calculated using the Mann–Whitney U test, where ** denotes a p-value < 0.0, and "n.s" denotes not significant.

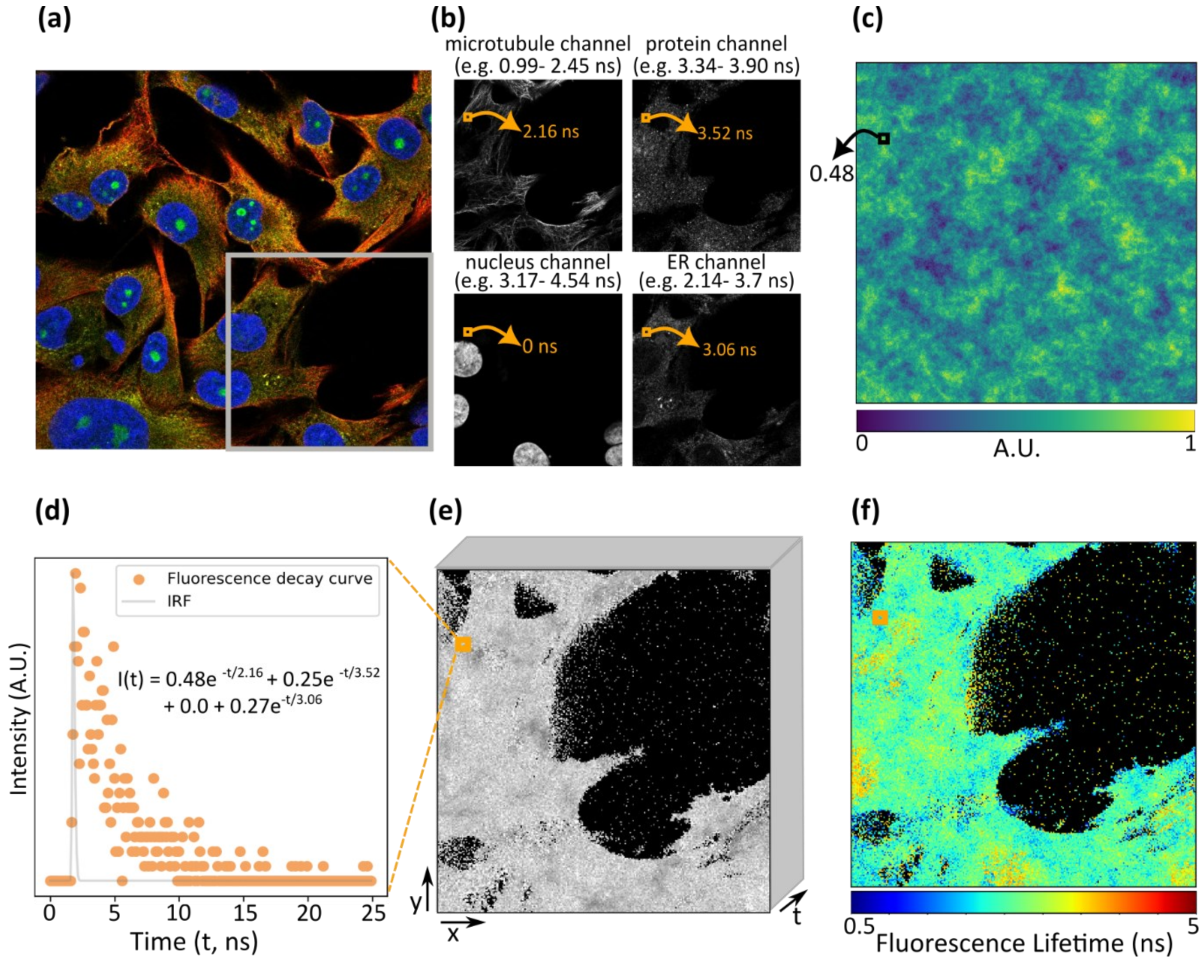

Supporting Figure 8. Methodology for simulating TCSPC-FLIM data using the HPA dataset[3]. (a) An example HPA image where the RGBY colour channels represent microtubules, protein, nucleus, and ER, respectively. A sliding window (highlighted in grey) is applied to extract sub-images with 256×256 pixels (x, y). **(b)** Each colour channel is randomly assigned a fluorescence lifetime range, as detailed in Materials and Methods. Example ranges are indicated above each image. For each colour channel, the fluorescence lifetime value for pixel i is displayed in orange. **(c)** Perlin noise is employed to determine the fractional contribution (a_i) of the first colour channel to each pixel. For example, for pixel i, $a_i = 0.48$. The contributions of the remaining colour channels to this pixel are then randomly assigned while ensuring that the total fractional contributions sum to 1. **(d)** Example fluorescence decay curve for pixel i generated using $I(t) = I_0 \sum_n a_n e^{-t/\tau_n}$, where I_0 is the initial intensity, a_n represents the fractional contribution of the n channel, and τ_n is its corresponding fluorescence lifetime. The calculation of $I(t)$ from the four channels at pixel i is shown. **(e)** Resulting 3D FLIM image where the x and y dimensions correspond to the composite intensity image of the four colour channels, while the t dimension contains fluorescence decay curves at each pixel. **(f)** Resulting fluorescence lifetime map where the location of pixel i is highlighted by the orange box.

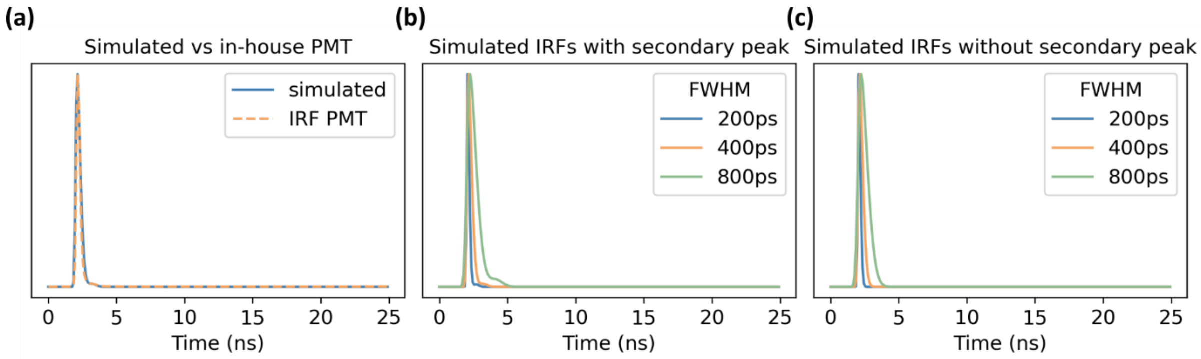

Supporting Figure 9. Simulated IRFs and their comparison to in-house data. (a) Comparison between an experimentally in-house acquired PMT IRF (orange) and a simulated IRF (blue). **(b)** Simulated IRFs with FWHM of approximately 200 ps (blue), 400 ps (orange), and 800 ps (green), with a secondary peak reflecting instrumental artefacts. **(c)** Simulated IRFs with FWHM of approximately 200 ps (blue), 400 ps (orange) and 800 ps (green), without the secondary peak.

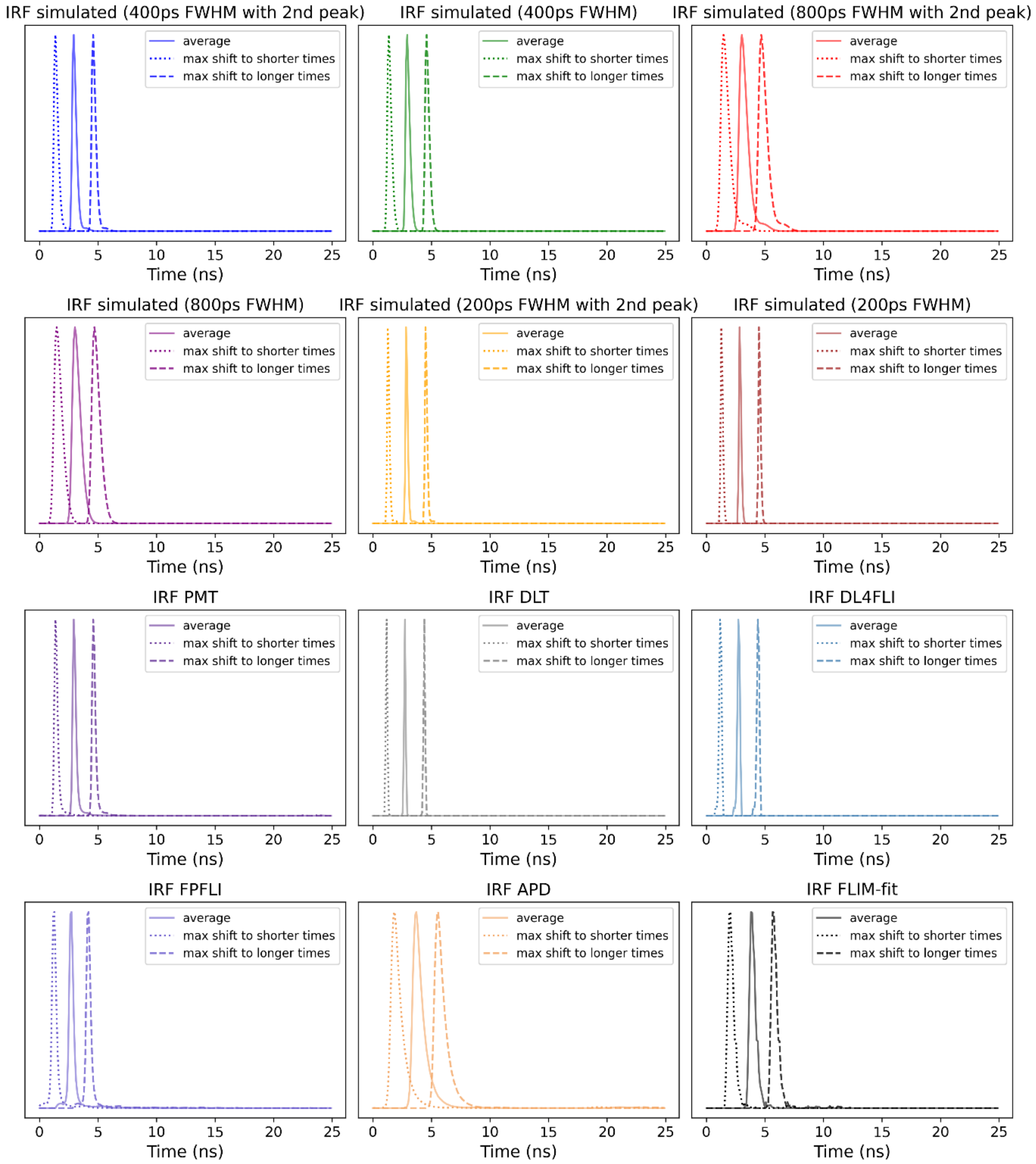

Supporting Figure 10. Complete set of IRFs used in the simulation of FLIM data. To replicate the laser jitter effect often observed in TCSPC-FLIM data, each IRF was randomly shifted to earlier or later time bins. This shifting simulates variability across different experimental setups, enhancing the generalisability of FLIMngo. For each IRF, the maximum shifts towards shorter and longer times are indicated by dotted and dashed lines, respectively, while the average peak position is shown as a solid line. The IRF peak positions spanned from the 12th to the 58th time bin.

References

1. Gao, D. *et al.* FLIMJ: An open-source ImageJ toolkit for fluorescence lifetime image data analysis. *PLoS One* **15**, (2020).

2. Smith, J. T. *et al.* In vitro and in vivo NIR fluorescence lifetime imaging with a time-gated SPAD camera. *Optica* **9**, 532 (2022).

3. Ouyang, W. *et al.* Analysis of the Human Protein Atlas Image Classification competition. *Nat Methods* **16**, 1254–1261 (2019).
